# Character Customization With Cosmetic Microtransactions in Games: Subjective Experience and Objective Performance

**DOI:** 10.3389/fpsyg.2021.770139

**Published:** 2022-01-04

**Authors:** Christian Böffel, Sophie Würger, Jochen Müsseler, Sabine J. Schlittmeier

**Affiliations:** Institute of Psychology, Work and Engineering Psychology, RWTH Aachen University, Aachen, Germany

**Keywords:** avatars, esports, games, human-computer interaction, identification, performance, customization, microtransactions

## Abstract

Free games that are monetized by selling virtual items, such as cosmetic microtransactions for one’s avatar, seem to offer a better gaming experience to paying players. To experimentally explore this phenomenon, the effects of character customization with cosmetic microtransactions on objective and self-estimated player performance, subjective identification with the avatar, fun and the players’ perceived competence were examined in the game League of Legends. This study introduces a new laboratory-based, experimental task to objectively measure within-game player performance. Each participant performed this game-based task in two different conditions: With a character that was customized using a provided set of cosmetic microtransactions and with a default character. Results showed that customization increased subjective identification with the player character. However, objective performance measures were unaffected by this manipulation although the novel experimental approach provided reliable performance results. Additionally, identification was positively related to perceived competence, fun, and self-estimated performance. Implications for the design of cosmetic microtransactions and their influence on competitive gaming are discussed.

## Introduction

Besides playing videogames recreationally, professional gaming and esports are attracting more and more attention. In esports it is increasingly important for developers, esports professionals and researchers to study possible determinants of performance. Among the competitively played games are so called *Multiplayer Online Battle Arena* (MOBA) games (Xia et al., 2019) like *League of Legends* or *DOTA2.* These games can be downloaded and played for free (free-to-play) but are monetized with microtransaction models that are successful and widely applied in the gaming industry. The system, in which players purchase virtual items through microtransactions, has been heavily criticized, because some microtransactions may provide players with competitive advantages (“pay-to-win”). Therefore, esports games often limit microtransactions to purely cosmetic changes (subsequently simply called *cosmetics*) to prevent competitive advantages for pay. However, it seems likely that cosmetics impact a player’s experience and – given the nature of human information processing – potentially even performance. Our aim was to investigate possible effects of cosmetics on players’ performance in a laboratory experiment. In addition, we aimed to examine experiential effects of cosmetics regarding a player’s identification with their avatar, experienced fun and perceived competence, as well as the interplay of these factors.

### Avatars and Customization

Cosmetics discussed here are applied to the controlled characters, so-called avatars, which act as the interface between the player and the virtual game world. Avatars are generally defined as virtual representations of the user. Selecting cosmetics in a game is a way to *customize* avatars and change the avatar’s appearance, for example, when choosing hair color (Ducheneaut et al., 2009). Customization strengthens the connection between the player and their avatar and increases identification with the avatar (e.g., Dolgov et al., 2014; Turkay and Kinzer, 2014). Cohen (2001) understands identification as the adoption of the identity and perspective of a character and it can occur cognitively, motivationally, empathetically, and as absorption (through loss of awareness of oneself). Different people create or chose avatars with different goals in mind. Neustaedter and Fedorovskaya (2009) identified four different types of users, such as “ideals” and “realistics” and Loewen et al. (2020) were able to link differences in avatar creation to differences between the real and ideal self. Another goal that influences avatar choice in a competitive setting is the need to overpower the opponent (Poeller et al., 2020).

Identification with the avatar increases intrinsic motivation (Birk et al., 2016) and might therefore also be relevant to player enjoyment and performance. The benefits of avatar customization for identification with the avatar are also relevant in digital mental health interventions and can improve intervention outcomes (Birk and Mandryk, 2019).

Avatars influence human perception and behavior fundamentally. Tajadura-Jiménez et al. (2017) showed that embodying a child-like avatar influences how a person estimates the size of an object and Böffel and Müsseler (2019b) demonstrated that avatars influence how the locations of objects are perceived. Some cosmetics also change the visual effects of abilities and such differences in action effects might impact behavior or the embodiment of the avatar (cf. Böffel and Müsseler, 2019a). Additionally, avatars and their characteristics influence complex behavior. One example is the *Proteus Effect* (for an overview see: Ratan et al., 2020). It describes the observation that users act in correspondence with the behavior they stereotypically associate with the appearance of their avatar, for example by negotiating more fiercely when they were represented by a taller avatar (Yee and Bailenson, 2007). Another striking example was reported by Yang et al. (2014) who showed that the avatar’s skin color can impact behavior and cause an increase in aggressive behavior after playing a violent game with a black avatar compared to playing the same game with a white avatar. Similarly, Hollingdale and Greitemeyer (2013) provided evidence that playing a customized avatar results in more aggressive behavior compared to playing a predetermined avatar. Changing the appearance of an avatar could be a viable tool to change a player’s behavior and effects of avatar appearance have been obtained for pro- and antisocial behavior (Yoon and Vargas, 2014), financial decision making (Hershfield et al., 2011) and even exercise habits (Fox and Bailenson, 2009). With regards to competitive games, choosing cosmetics that look particularly powerful might cause players to try harder or playing with your favorite skin could increase performance as a result of increased intrinsic motivation (cf. Birk et al., 2016).

In summary, the interaction between a person and their avatar seems bidirectional: The person changes their avatar, but their avatar might also change the person’s behavior in return. This relationship could potentially encompass performance relevant aspects of player behavior and our goal in this study is to investigate this in a MOBA game.

### Research Questions

Our goal in this study was to examine the impact of selecting cosmetics on performance and subjective experience in a game. First, we test the hypothesis that playing with customized avatars leads to increased identification (Hypothesis 1). Second, we expected that playing with a customized avatar leads to increased fun (Hypothesis 2). Third, we investigate the hypothesis that customization increases the perceived competence of players (Hypothesis 3) as the customization process increases interactivity. In addition, we wanted to examine whether a player’s self-estimated and actual performance is affected by customization. Lastly, we explore the correlations between these measures of experience and performance.

## Materials and Methods

### Participants

A total of 24 participants (14 female, 10 male) aged 19–32 years (*Md* = 22.0) participated voluntarily and in some cases for course credit. The sample allowed to detect effects of medium sizes (*d* = 0.52) for the within-subjects *t*-tests with a power of β = 0.8. The participants stated in a preliminary survey that they played a median hours of video games of *Md* = 5.0; range: [0;30] and six participants did not play any video games at all. Eight stated to regularly play MOBA-type games like League of Legends, sixteen participants reported to not play the game. Participants who played League of Legends reported a median of *Md* = 7.0; range: [1;25] hours per week.

### Material

We used an experimental setup using the practice tool in the MOBA game *League of Legends* to create a highly controlled situation suitable for experimental testing. This simplification of the gameplay ensured that inexperienced players could participate and increased comparability between measurements by preventing the introduction of additional disturbances, e.g., from other players. The participants could choose between a male and a female character to account for possible gender differences regarding the avatar’s gender choice to maximize identification. We used the game character “Ezreal” as the male and the character “Lux” as the female avatar because both are easy to control ranged fighters with human appearance and a suitable set of cosmetics. In the choice condition, participants were able to choose between eight cosmetics for each avatar and the default appearance (Supplementary Appendix B). In the no-choice condition, only the default appearance of the avatar was used. For both characters, the cosmetics available covered different potential goals of customization. Some cosmetics had a powerful and serious appearance, while others were rather humorous in nature. This way, we hoped to offer each participant a choice of cosmetics that fit their own personal preference.

### The Gaming Task

**FIGURE 1:**
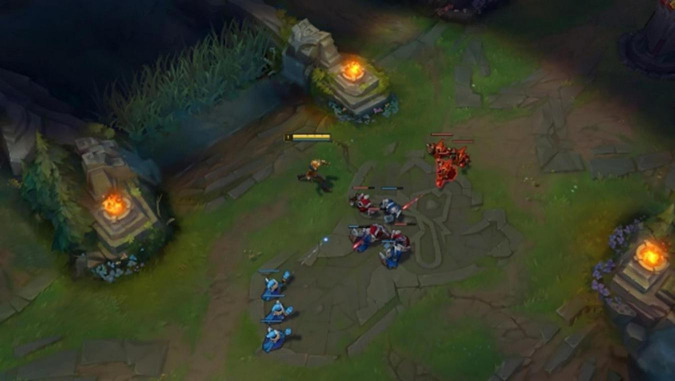
Players’ view of the game with the male avatar and default appearance. The allies (blue) and enemies (red) automatically attacked each other. The player’s task was to score a last hit on the enemies, the final hit that causes the enemy to be defeated. Images reproduced with permission from the League of Legends Brand Manager DACH.

The participants controlled the avatar by right clicking on the game map and they were asked to position their character in the middle of the map (Figure 1). A series of small allies and enemies appeared that started attacking each other. The participants’ task was to attack enemies at the right time to defeat the enemy. Only if they secure the final attack on the enemy, the attack is successful, resulting in a *last hit*. Last hits are an important aspect of performance in League of Legends as they provide money that is used to buy items and improve the avatar’s strength (Leavitt et al., 2016). To be successful in this task, timing is crucial. If the participant attacks too late or too early, the enemy is defeated by other allies and the *last hit* is lost. Attacks could be triggered by right clicking and participants were only allowed to use these basic attacks. The participants used headphones during the task. The game settings were kept as simple as possible and constant for all participants and conditions. The in-game camera was fixed on the avatar (Figure 1). Each task round lasted 10 min and the timer was started as soon as the first enemies arrived at the players’ position in the middle of the map. After each round, the amount of last hits was recorded as an objective measure of performance.

### Measures

We created a questionnaire using SoSci Survey (Leiner, 2019) to quantify the participants’ subjective experiences (Supplementary Appendix A). All items were used in a German translation and the participants were asked to state their agreement to them on a six-point Likert scale from 1 (not at all) to 6 (very) unless otherwise specified. The questionnaire was kept short and simple in order to make sure that participants respond to the items as soon as possible after playing the game.

We measured *identification* with four items based on an identification scale by Schneider (2004). The item “I was interested in my character’s goal in this game” was replaced by the self-developed item “I liked the look of the character,” because the participants were uninformed about the character’s goals.

To measure *fun*, we used a combined scale of two items from each of the “positive affect” and “negative affect” scales of the GEQ Core Module by Ijsselsteijn et al. (2013).

*Perceived competence* was measured using a scale which consisted of three items of the “Competence” scale of the GEQ Core Module (Ijsselsteijn et al., 2013) and one additional item regarding perceived control over the character. The internal consistency of these scales were good in each of the two measurements with α (first) = 0.850 and α (second) = 0.781 for the first and second measurement of identification, α (first) = 0.852 and α (second) = 0.782 for fun, and α (first) = 0.816 and α (second) = 0.870 for perceived competence.

As a measure of *estimated performance*, participants were asked to estimate their last hits with a slider from 0 to 100%.

In addition to these subjective measures, we used the amount of last hits as an objective measure of performance. A maximum of 126 last hits was the theoretical performance maximum in this game-based task and to increase comparability to the participants’ self-estimates, we calculated the percentage of last hits. The correlation of last hits between both conditions for each participant was high with *r* = 0.783.

### Procedure

**FIGURE 2:**
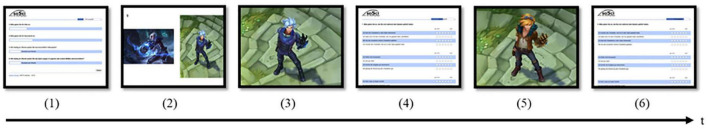
Visualization of the process for participants who first played with cosmetics: (1) preliminary interview, (2) cosmetics selection, (3) practice round plus 1st game round with cosmetics, (4) 1st questionnaire, (5) 2nd game round without cosmetics, and (6) 2nd questionnaire. Images reproduced with permission from the League of Legends Brand Manager DACH.

The experiment was carried out in a laboratory. After the participants gave their written informed consent in accordance with the Declaration of Helsinki (World Medical Association, 2013), they were introduced to the game mechanics and asked to choose the gender of their avatar. Then, a short preliminary survey was conducted to collect demographic data and previous experience with video games and MOBAs. The order of the two conditions (no choice and choice) was counterbalanced between participants. In the choice condition, participants selected their preferred cosmetics from a set of nine slides. The choice of cosmetics was relatively evenly distributed (for the exact distribution see Supplementary Appendix B). In the no-choice condition, participants used the avatar’s default appearance. All participants completed a short practice phase to familiarize themselves with the controls. Then, the first 10-min round of the game-based task was carried out and the amount of last hits was recorded before the participants completed the subjective experience questionnaire. Then, the second 10- min round of the remaining condition was started and performance was recorded. At the end, the participants answered the same questionnaire again. In addition, after both rounds were completed, an open question asked why participants chose their cosmetics (Supplementary Appendix C). It took participants about 45 min to complete the experiment. An overview of the course of the experiment is shown in Figure 2.

### Design

The condition choice (customized with cosmetics) vs. no-choice (default appearance) was used as an independent within-participant variable. Identification, perceived competence, estimated last hits, and fun as subjective measures were measured *via* questionnaires. The number of last hits was used as an objective performance measure.

## Results

### Effects of Customization

Differences between the choice and no-choice condition were tested using paired samples, two-tailed *t*-test and Cohen’s *d* is given as an effect size measure. The English translation of the items is shown in Supplementary Appendix A.

**FIGURE 3:**
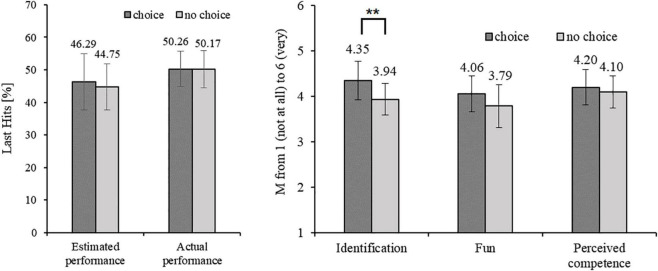
The self-estimated and actual performance operationalized as percentage of last hits, and mean values from 1 (not at all) to 6 (very) of the collected variables identification, fun and perceived competence in conditions with and without choice. The error bars represent the 95% confidence intervals. *p < 0.05, **p < 0.01.

The self-estimated and actual performance during the two different customization conditions is shown in Figure 3 (left). There was no significant difference between conditions neither for self-estimated performance, *t*(23) = 0.732, *p* = 0.472, *d* = 0.149 nor for actual performance, *t*(23) = 0.67, *p* = 0.947, *d* = 0.014.

The differences between the choice and no-choice condition in identification, fun and subjective performance are shown in Figure 3 (right). Identification was significantly higher in the choice condition compared to the no-choice condition, *t*(23) = 3.391, *p* = 0.003, *d* = 0.692, indicating that playing with a customized avatar increases identification (H1). However, playing with a customized avatar did not increase fun (H2), *t*(23) = 0.732, *p* = 0.472, *d* = 0.230, or perceived competence (H3), *t*(23) = 0.792, *p* = 0.436, *d* = 0.162.

### Correlations

To further explore the relationships between the individual variables, repeated measures correlations of the dependent variables were analyzed using the R package “rmcorr” (Bakdash and Marusich, 2017).

As shown in Table 1, identification was positively and significantly associated with all other measures of subjective experience. Additionally, competence and estimated last hits were significantly related, which is to be expected. Estimated and actual performance were positively associated, meaning that participants were able to rate their performance with some degree of precision. Furthermore, actual performance had no significant correlation with fun or identification.

**TABLE 1:**
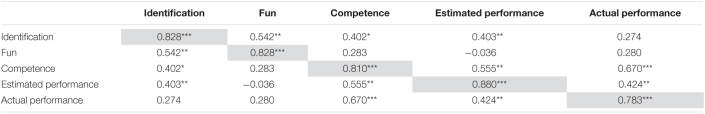
Repeated measures correlations of the dependent variables.

The diagonal shows correlations of each variable between both measurement points. *p < 0.05; **p < 0.01; ***p < 0.001.

## Discussion

This study examined how cosmetic customization of avatars affects the gaming experience and the performance in the game using a novel experimental approach. Participants performed a task within League of Legends, with or without choosing cosmetics. We analyzed how this manipulation affected avatar identification, fun, perceived competence, and estimated and actual performance and how these measures were related to each other.

The actual performance was not significantly influenced by customization and the high correlation of performance between the choice and no-choice condition shows that last hits are a reliable measure of performance. We therefore found no evidence that the customization of avatars influenced the performance in this game-based task. Whether this result can be applied to other performance aspects or a player’s overall performance in a competitive match should be further investigated as customization could change other cognitive aspects. The use of certain cosmetics could influence cognitive processes. For example, eye-catching cosmetics might cause an attention shift toward the avatar. Such an attention shift could be detrimental to performance as it potentially interferes with the participant’s ability to monitor the enemies, which is necessary for a good performance in this task. Importantly, since there was no significant difference in performance between both conditions, we can likely exclude the possibility that performance impacted the observed effects of subjective measures.

Regarding the subjective measures of experience, we were able to confirm Hypothesis 1 — the assumption that customization of the avatar increases identification. This result is in line with earlier studies (e.g., Dolgov et al., 2014) and shows that choosing cosmetics is a suitable way to customize avatars. One possible explanation is that the choice condition increased the perceived interactivity of the game, because it allows for more control over the appearance of the game. This interactivity could then lead to increased identification (Hefner et al., 2007).

Interestingly, we found a positive relationship between identification and fun, similarly to Trepte and Reinecke (2010), but this connection did not lead to a significant difference in fun between the choice and no-choice conditions. Even though we descriptively observed more fun in the choice condition, Hypothesis 2 needs to be rejected. An increased identification with the avatar could facilitate escapism from reality (Yee, 2006) and thus lead to more fun (Henning and Vorderer, 2001). However, other differences between the choice and no-choice conditions might have suppressed the positive influence of increased identification in the choice condition. It is, for example, possible that participants disliked the process of selecting the cosmetics itself. One reason for this could be the laboratory setting and the presence of the examiner. Participants might have felt judged for their choices which could have caused discomfort and they might not have chosen the most fun cosmetics. Such effects of reactivity might also explain why male participants chose female avatars less often than expected based on previous research. For example, Ratan et al. (2019) found that women choose a gender-consistent character more often compared to men. However, in the current study, 64% of the women and 70% of the men chose characters of their own gender.

The results also showed that the customization of avatars did not affect perceived competence, we therefore need to reject Hypothesis 3. However, we observed that perceived competence is related to identification, a connection that was previously identified by Klimmt and Hartmann (2006). In fact, identification correlates more strongly with perceived competence than with actual performance. On the one hand, increased identification with the avatar could lead to the feeling that the avatar is easier to control, which in turn would lead to the perception of increased competence. On the other hand, the increased perceived competence and associated experience of self-efficacy (Bandura, 1977) could trigger an increased identification with the avatar, since it might be easier to identify with the avatar if the player has the feeling of control. This assumption is in line with previous research that demonstrated that a higher degree of control or agency in the person-avatar interaction increases subjective measures of the person-avatar interaction related to identification, such as perceived body ownership of the avatar (Böffel and Müsseler, 2018, 2019a).

### Limitations

While the game task we used has several advantages and proved useful in a lab setting, it is also associated with several limitations that need to be discussed. For example, the sample size that is common for laboratory testing could be insufficient to detect smaller effects that might be present, e.g., the influence of customization on fun and the power analysis showed that only medium sized effects can be reliably detected by this design. For the same reason, some correlations that were not significant in this study should not be dismissed outright but instead investigated further, such as a possible connection between fun and performance. Furthermore, perceived competence and estimated last hits were only moderately correlated, which indicates that only some aspects of subjective performance are represented in the last hit measurements. This corresponds to the fact that the task only represents a certain performance aspect. However, performance was related to perceived competence and this may indicate that participants were able to accurately judge their competence and that their perceived competence is grounded in objective measures.

Additionally, the measures we used were short and while we chose items that have a high face validity, our questionnaire has not been subjected to an extensive analysis of validity. Given the limited sample size, factor analysis and similar analyses were not an option, and it is possible that the structure of the questionnaire is different to what we expected.

Even though we used a repeated measures design to control for different levels of skill and experience, the differences between participants in terms of prior exposure to the game could have impacted our task. The relationship between performance and identification could, for example, be influenced by previous experience with the game in a way that only players with limited experience benefit from customization. Effects of identification could be limited by a ceiling effect of performance on one hand, and on the other hand, the impact of increased identification through customization could be enhanced by the novelty of customization, experienced only by novice players. Experienced players also know that the task only featured a limited number of aspects when compared to actual competitive gameplay. Additionally, prior experience might have influenced the choice of cosmetics because some participants stated that they had chosen cosmetics because they had never played them before.

Furthermore, the choice of the avatar’s gender could have had an influence on the collected data as it could itself satisfy the want for customization and therefore limit the impact of choosing cosmetics due to ceiling effects. Thus, it is possible that gender selection is the reason why the non-customized condition does not have such different results compared to the customized condition.

### Implications

The present study shows that personalizing avatars improves identification with them and that fun, subjective competence and estimated performance are linked to identification. Regarding competitive gaming, this study showed that the influence of selecting cosmetics is likely negligible for performance in the examined task. However, the task is primarily a motor task that focuses on performing precise mouse-actions with the correct timing and further studies are needed to determine whether other aspects of performance, e.g., communication with team members or complex decision making is also unaffected by selecting cosmetics. They could also use a more complex setting to account for the high complexity in competitive gaming. Especially social factors, which are a crucial reason why cosmetics are bought (Kordyaka and Hribersek, 2019) should be taken into account by integrating other players into the task. The absence of a competitive, social situation could also explain the lack of a connection between identification and fun as it is expected that showing off a flashy skin is an enjoyable experience as it demonstrates the player’s status. Additionally, it is important to note that the cosmetics in our study were not earned or bought by the participants, which could be a factor that also influences fun and possibly even performance through differences in player motivation. It is possible that the perceived enjoyment to use self-bought cosmetics increases in order to justify the decision to spend money on them according to the theory of cognitive dissonance (Festinger, 1962).

Furthermore, the results could be relevant in other contexts, e.g., serious games. Serious games are used in various areas such as school or health care. They are valuable tools to teach things such as mathematics (Alexandrovsky et al., 2021) and can be used to increase participants’ motivation in gamified tasks used in cognitive assessments (Friehs et al., 2020). Serious games often feature avatars and Oksanen et al. (2013) showed that increased identification with the avatar improves learning. The effects of customization might also differ between older and younger people as they show different priorities and aims while playing and as a result, customization could be more important for older players who show a focus on choice and enjoyment (Birk et al., 2017). Based on our results, the option to personalize avatars could have a positive effect on learning from serious games as well, as it increases identification.

Furthermore, it would be interesting to examine the role of self-efficacy (Bandura, 1977). Trepte and Reinecke (2011) asked participants to play a jump and run game and recorded their enjoyment, self-efficacy, and performance. They were able to demonstrate that performance and fun were associated with enjoyment and that the relationship between performance and enjoyment was mediated by self- efficacy. The perceived interactivity of the game has also already been investigated as a relevant factor for the gaming experience (e.g., Jin, 2009) and could be included as a possible mediator in future research.

## Data Availability Statement

The raw data supporting the conclusions of this article will be made available by the authors, without undue reservation.

## Ethics Statement

Ethical review and approval was not required for the study on human participants in accordance with the local legislation and institutional requirements. The patients/participants provided their written informed consent to participate in this study in accordance with the Declaration of Helsinki (World Medical Association, 2013) and participation was voluntary. Further, no undue physical or psychological stress by participating in this study was anticipated and participants did not take risks by participating. Participants were fully informed about the aims and procedures of this study and could withdraw their participation any time. As a result, no ethical concerns were identified in accordance to the ethics guidelines of the DFG (Deutsche Forschungsgemeinschaft [German Research Foundation], 2021).

## Author Contributions

CB conceived the initial idea and wrote the manuscript with the aid of an initial draft of SW and input from all authors. CB and SW designed the study and contributed to the data analysis. SW collected the data under the supervision of CB. SW, JM, and SS provided helpful comments and improvements to the manuscript. JM acquired funding for this study.

## Conflict of Interest

The authors declare that the research was conducted in the absence of any commercial or financial relationships that could be construed as a potential conflict of interest.

## Publisher’s Note

All claims expressed in this article are solely those of the authors and do not necessarily represent those of their affiliated organizations, or those of the publisher, the editors and the reviewers. Any product that may be evaluated in this article, or claim that may be made by its manufacturer, is not guaranteed or endorsed by the publisher.
