# Supplementary material for: Character Customization With Cosmetic Microtransactions in Games: Subjective Experience and Objective Performance
Source: Front Psychol. 2022 Jan 4;12:770139. doi: 10.3389/fpsyg.2021.770139 (PMC8765231; doi:10.3389/fpsyg.2021.770139)
Supplement: Supplementary file 1 [file Presentation_1.pdf]

Appendix A

Items used to collect the subjective data

Identification

1. I felt the characters in this game were interesting
2. I identified with the character I was playing in this game
3. I liked the character I was playing in this game
4. I liked the look of my character

Perceived competence

1. I felt competent
2. I was good at it
3. I was able to complete the task well
4. I managed to control the character well

Estimated performance

1. What percentage of opponents do you think you could hit with a "last hit"?

Fun

1. I thought it was fun
2. It gave me a bad mood (-)
3. I felt bored (-)
4. I enjoyed it

Note.. Items marked with (-) have negative polarity.

Appendix B

Frequency of the selected cosmetics for the female character Lux and the male character Ezreal in the choice condition

Lux (without skin)	1	Ezreal (without skin)	1
Academia	2	Academia	1
Pajama Guardian	2	Pajama Guardian	2
Elementalist	1	Star Guardian	2
Star Guardian	0	Arcade	0
Steel Legion	2	Ace of Spades	1
Commando	0	Debonair	0
Spellthief	4	Pulsefire	3
Sorceress	0	Frosted	2
∑ female	12	∑ male	12

Appendix C

Frequency of reasons for selecting cosmetics as indicated in an open answer format. Multiple answers were possible.

appealing look	10
interesting/mysterious look	6
strong look	4
Experience based (new skin/expensive etc.)	4
funny/cute	3
similar to me	2
first skin in order	1
